# Influences of semantic and syntactic incongruence on readiness potential in turn-end anticipation

**DOI:** 10.3389/fnhum.2014.00296

**Published:** 2014-05-27

**Authors:** Hendrik Wesselmeier, Stefanie Jansen, Horst M. Müller

**Affiliations:** Experimental Neurolinguistics Group, Collaborative Research Center “Alignment in Communication” (SFB 673), Bielefeld UniversityBielefeld, Germany

**Keywords:** turn-end anticipation, spoken language, EEG, syntactic errors, semantic errors, readiness potential

## Abstract

Knowing when it is convenient to take a turn in a conversation is an important task for dialog partners. As it appears that this decision is made before the transition point has been reached, it seems to involve anticipation. There are a variety of studies in the literature that provide possible explanations for turn-end anticipation. This study particularly focuses on how turn-end anticipation relies on syntactic and/or semantic information during utterance processing, as tested with syntactically and semantically violated sentences. With a combination reaction time and EEG experiment, we used the onset latencies of the readiness potential (RP) to uncover possible differences in response preparation. Although the mean anticipation timing accuracy (ATA) values of the behavioral test were all within a similar time range (control sentences: 108 ms, syntactically violated sentences: 93 ms and semantically violated sentences: 116 ms), we found evidence that response preparation is indeed different for syntactically and semantically violated sentences in comparison with control sentences. Our preconscious EEG data, in the form of RP results, indicated a response preparation onset to sentence end interval of 1452 ms in normal sentences, 937 ms in sentences with syntactic violations and 944 ms in sentences with semantic violations. Compared with control sentences, these intervals resulted in a significant RP interruption for both sentence types and indicate an interruption of preconscious response preparation. However, the behavioral response to sentence types occurred at comparable time points.

## INTRODUCTION

**FIGURE 1:**
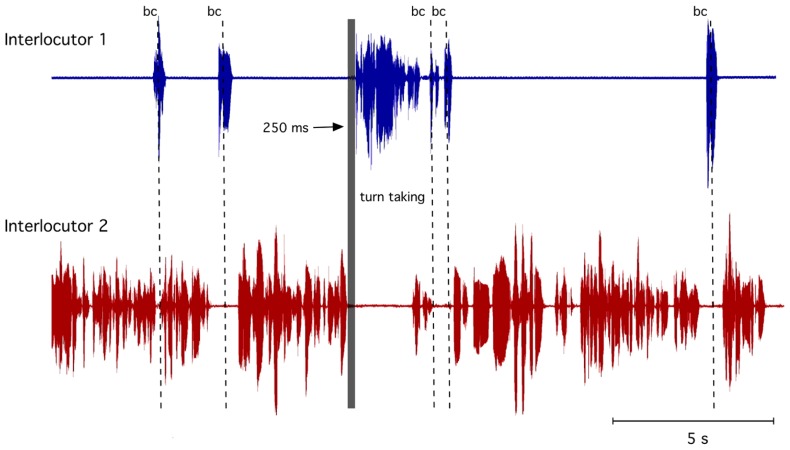
**Example of a dialog with accurate turn-taking (within 250 ms) indicated by the gray bar and backchannel (bc) indicated by the dotted lines**.

Human communication usually occurs with a bi-directional information exchange, which can be performed coevally or alternately. An example of simultaneous communication would be “mimic signals” (non-verbal) in which communication partners are simultaneously smiling. In the visual modality, a smile can be answered with a smile while the communication partner is still smiling, i.e., the smiles can overlap without interfering with each other. However, in a more complex information exchange such as spoken language, a sequential organization of the signals is required. In contrast to non-verbal signals, it is socially restrictive not to have two interlocutors speaking at the same time because speaking and listening simultaneously is not easily possible. Indeed, in a conversation, we need to process and understand a perceived utterance before we can react by replying. Thus, in conversations between two or more interlocutors, the organization of taking turns is fundamental such that predominately only one partner speaks at a time. Sacks et al. (1974) already demonstrated that conversationalists tend to avoid silences and overlapping speech. To achieve this highly coordinated timing, we need to know in advance when our interlocutor will finish his turn to prepare our response. Many studies have attempted to explain this ability and have formulated a number of hypotheses about the information that listeners use to anticipate utterance endings (e.g., Grosjean, 1996; Magyari and De Ruiter, 2012). Because the length and content of what is said in a turn is not specified in advance, this information must be determined by the interlocutor during the turn itself. The complex task of determining the next transition-relevant place and preparing what to say requires highly synchronized turn-taking to ensure that virtually no gap will occur when the next speaker begins to speak. For example, this highly precise interplay can be observed in two phenomena: back-channeling and turn-taking. Back-channeling is the precise use of brief interjections (e.g., “aha” or “yeah”) that are not treated as an attempt to take a turn but rather used to signal attention, understanding and assent. They can occur in the short gaps within the interlocutor’s turn but also can overlap. For turn-taking, however, the decision concerning when to speak depends on several higher cognitive and motivational considerations. **Figure 1** shows an example of back-channels and turn-taking in a natural dialog.

Turn-taking may depend on situational factors, such as the relevance and pragmatic necessity of a possible utterance, how confident the interlocutors are about the reliability of the information, and social status of the interlocutors. However, Wilson and Wilson (2005) claim the precise timing of the speech onset at the moment the speaker begins to speak is mechanistically governed.

Turn-taking appears to be robust across different languages (Stivers et al., 2009). Infants develop turn-taking ability well before language acquisition (Beebe et al., 1988), suggesting that turn-taking ability is independent of linguistic development (Yoshida and Okanoya, 2005). However, early turn-taking ability is unlikely to be influenced by syntax within an utterance or the semantics of a message. Nevertheless, both of these factors are needed to form a correctly communicated illocutionary act. To prepare their response and to take their turn at the right moment, listeners need to know in advance when the current turn is going to end. What type of information do listeners base their knowledge of a turn-end on? The aspects under discussion are intonation (e.g., Caspers, 2003), prosody (e.g., Wells and Macfarlane, 1998), syntactic and semantic information (e.g., Sacks et al., 1974; De Ruiter et al., 2006) and pragmatic information (e.g., Ford and Thompson, 1996).

To test the possible influences on turn-end anticipation, De Ruiter et al. (2006) manipulated the presence of the symbolic (lexico-syntactic) content of utterances recorded in natural conversations. With non-recognizable words, participants’ button-press reaction-time increased significantly. Their experimental results revealed that the lexico-syntactic content of an utterance is necessary for successful turn-end anticipation. Magyari and De Ruiter (2008) attempted to determine how interlocutors use the lexico-syntactic information to anticipate the ends of turns so accurately. They hypothesized that listeners can predict when a turn is going to end because they can predict how it ends. Using a gating task, they were able to demonstrate that participants were best at predicting the final two words of those turns that were anticipated most accurately in a previous experiment by De Ruiter et al. (2006).

Contrary to anticipation in turn-taking, a fast reaction of the listener can offer an alternative explanation for turn-taking. Heldner and Edlund (2010), for example, investigated turn-taking based on the analysis of three conversational corpora from Dutch, Swedish, and Scottish-English. Their results indicated that turn-taking is less precise and more distributed than often claimed. With 40% of their material exceeding a 200 ms gap as the minimal response time, they concluded that reaction could be used as a model for timing in turn-taking. In contrast, an MEG experiment from Kuriki et al. (1999) found speech-related activity from the left frontal area of subjects 120–320 ms before the speech onset. In their task, subjects were asked to count numbers silently, while at a random time, a visual cue appeared, after which the subjects counted the numbers aloud. As this pre-activation for word production is highly unlikely to occur before gap detection is completed, it can only follow gap detection. Thus, these 120–320 ms pre-activations for word production result in a 200 ms gap as the minimal response time being insufficient for the reaction model because even the minimum of a 120 ms pre-activation prior to word production would reduce a 200 ms gap as the minimal response time to 80 ms for gap detection. In the reaction model (Heldner and Edlund, 2010), not taking higher cognitive processes for utterance preparation into account, a 120 ms pre-activation during word production can only start after 200 ms for minimal gap detection. In other words, when turn-taking with gaps under at least 320 ms, listeners must be able to anticipate when the upcoming transition relevant place will occur to prepare their response. Given these conflicting findings that speak for and against the general perspective of anticipation in turn-taking, further investigation is required.

Turn-end anticipation is usually measured by behavioral responses, for instance, anticipation timing accuracy (ATA), which reflects conscious behavioral processes. The ATA is the accuracy of the interval from the sentence-offset to response. However, the possible similar ATA results between conditions can nevertheless fail to reflect the operation of different anticipatory performances, as compensated for by short response-preparation in late anticipation and longer response preparation in early anticipation. The current EEG experiment was conducted to test the usability of readiness potential (RP) as a neurocognitive preconscious measure during turn-end anticipation and to test the influence of syntactic and semantic violations on turn-end anticipation performance based on the RP as a preconscious measure. To investigate the RPs, our experiment used acoustically presented normal utterances (controls) and utterances containing a syntactic or a semantic violation. The forms of violations were selected as possible key factors for successful language comprehension.

Even though independent sentences without context as a global semantic component might appear to be too unlike natural turn-end anticipation, Magyari and De Ruiter (2012) demonstrated in their experiment that button presses for out-of-context turns are also accurate. To obtain a better understanding of the temporal process of response preparation in turn-end anticipation, we consulted the RP as a well-established neuronal correlate in response preparation. The RP was first described by Kornhuber and Deecke (1965) and is assumed to be related to selective response activation processes for hand movements. The RP is most present (Kutas and Donchin, 1974) and maximal (Deecke et al., 1969) over the contralateral motor cortices of the contrasting hand obtained at the C3 and C4 electrodes as specified by the 10/20 system (Jasper, 1958). The RP is presumed to reflect the average amount of brain activation related to the motor preparation of the responding hand. Assuming that people prepare to respond as soon as they have an expectation when the sentence will end, an RP indicating preparation should develop. An earlier experiment (e.g., van Turennout et al., 1998) has already demonstrated that the RP is sensitive to language comprehension. In their experiment, Dutch participants saw pictures of objects and had to perform a go/no-go task in which they had to push a button with their left index finger for objects with a common gender and push a button with their right index finger for objects with a neuter gender. In addition, they only had to push the button (go) for objects with an initial “b” and not to push (no-go) for objects with an initial “s.” With this paradigm and their RP results, they concluded that syntactical information of the object is retrieved 40 ms before phonological information is retrieved. Basically, findings such as this demonstrate the sensitivity of the RP as a suitable measure of brain activity for studying the time course of encoding various levels of information and capturing the time course of language processing during response preparation.

To investigate turn-anticipation rather than turn-taking, we used button presses as responses to eliminate confounding variables of verbal responses, e.g., the breathing in before verbalization, the preceding higher cognitive processes for verbal response preparation and the motor complexity of verbalization. Based on previous research, two questions arise: (1) Is it possible to find response-related electrophysiological correlates providing strong evidence for early turn-end anticipation, and is the RP onset a suitable measurement that can be used to find differences in turn-end anticipation? (2) If turn-end anticipation is different for violated sentences, would syntactic incongruence, semantic incongruence or both affect turn-end anticipation?

## MATERIALS AND METHODS

### PARTICIPANTS

A total of 30 students (17 women and 13 men) between 19 and 35 years of age (Ø = 24.5 ± 3.5 years) were recruited from Bielefeld University. All subjects were native German speakers and right-handed with a lateralization quotient of 88.9 according to the Edinburgh Handedness Inventory (Oldfield, 1971). According to their own accounts, the participants did not suffer from any auditory or motor restrictions or diseases that could have influenced the results. Each subject participated in a single session lasting 1.5–2 h. Written informed consent was obtained from all participants, and the study was approved by the Ethics Committee of Münster University.

### STIMULI

The stimuli in this experiment consisted of 184 sentences, of which 69 critical sentences were matched between the conditions with psycholinguistic criteria (sentence length (see **Table 1**), number of words (Ø = 14.2 ± 2.4 SD), number of syllables (Ø = 22.5 ± 3.1 SD), and syntactic complexity and affectivity) along 115 non-matched filler sentences. The filler sentences consisted of a variety of syntactic structures, whereas critical sentences consisted only of a slightly deviating syntactic structure. All sentences in the experiment were spoken at a speed of 400–450 syllables per minute by a professional female speaker with natural intonation and recorded in a sound studio. The 69 critical items consisted of 23 control sentences, 23 sentences with a syntactic violation, and 23 sentences with a semantic violation. All critical sentences were pseudo-randomized among the 115 filler sentences. For this within-subject design, three groups were created in a Latin square design to ensure that the participants would not hear the same sentence from different conditions. Therefore, the participants heard at least seven sentences from each condition along with all filler sentences. The overall sentence length of all conditions varied from 3287 to 5803 ms, whereas the filler sentences varied from 1097 to 6643 ms (see **Table 1**). The time between the end of a critical word and the sentence end varied between 863 and 2123 ms (see **Table 2**). Examples from the stimuli can be seen below. A # indicates the moment at which the violations end.

**Table:** 

Control:	Der Pfarrer hatte stets dreimal die Glocke geläutet# bevor er zum Essen ging.
	*(The priest always rang the bell three times before he went to dinner.)*
Syntactic:	Der Pfarrer hatte stets dreimal die Glocke läuten# bevor er zum Essen ging.
	*(The priest always rings the bell three times before he went to dinner.)*
Semantic:	Der Pfarrer hatte stets dreimal die Glocke gegrinst# bevor er zum Essen ging.
	*(The priest always grinned the bell three times before he went to dinner.)*
Filler:	Selbst die Großeltern hatten auf dem Jahrmarkt eine Menge Spaß.
	*(Even the grandparents had a lot of fun at the funfair.)*

**Table 1:** Mean articulatory length of the 184 stimuli used in the experiment.

Condition	Mean (ms)	Min (ms)	Max (ms)	SD
Control	4472.7	3287	5619	619.8
Syntactic	4483.0	3485	5688	680.1
Semantic	4447.3	3618	5803	702.6
Filler	3513.7	1300	6643	1112.5

*The articulatory length did not differ significantly between the three critical conditions [*F*(2, 66) = 0.017, *p* = 0.983].*

**Table 2:** Mean articulatory length of the clauses critical word-end to stimulus-end.

Condition	Mean (ms)	Min (ms)	Max (ms)	SD
Control	1348.6	885	1871	285.8
Syntactic	1385.0	876	2123	301.2
Semantic	1371.0	863	2038	292.0

*The articulatory length of the clauses critical word-end to stimulus-end did not differ significantly between the conditions [*F*(2, 66) = 0.089, *p* = 0.915].*

### PROCEDURE

Participants were seated comfortably in an armchair with a USB button-box for the button press in their right hand. They were instructed to place their arms and hands on the elbow rest and hold their index finger on the response button. The participants sat in front of a computer screen with external speakers. Each trial started with a fixation cross presented in the middle of the screen from 3.8° to 0.2° of visual angle. After the fixation cross appeared, the stimulus sentence started after a random inter-trial interval (range 1000–2500 ms) so that the participants could not anticipate the sentence onset time after several trials. The fixation cross was continuously shown until at least 1000 ms after the sentence ended. The mean stimuli intensity ranged between 55 and 60 dB, which matches a normal face-to-face conversation. During the EEG recording, the participants listened to the sentences, and they were instructed with the following wording: “Try to push the button exactly when the sentence ends. Do not wait until the sentence has ended definitely but rather push the button at the time point you would expect its end.” After a short practice block with 20 non-critical sentences, all participants felt comfortable with the anticipation task, which was introduced in De Ruiter et al. (2006).

### RECORDING

The EEG recording took place in a sound-proofed and electromagnetically shielded booth. The EEG was continuously recorded from 32 active scalp electrodes (ActiCap, Brain Products Inc.) placed at locations based on the International 10/20 system with reference at FCz. Horizontal eye movements were recorded from the left and right outer canthi (hEOG), and vertical eye movements were recorded from above and below the right eye (vEOG). The signals were sampled at 1000 Hz, amplified with a bandpass of 0.16–80 Hz and a 50 Hz notch filter by amplifiers (QuickAmp, Brain Products Inc.) and recorded with BrainVision Recorder software (Version 1.20, Brain Products Inc.). The impedance was kept below 5 kΩ for all channels prior to recording. A button press was recorded by a USB button-box with an internal clock.

### ANTICIPATION TIMING ACCURACY ANALYSIS

Anticipation timing accuracy data analyses were conducted using EEGLAB (Delorme and Makeig, 2004) under MATLAB for Linux. First, a marker-table was exported for each participant for ATA analysis. Statistical analyses and calculations were performed via SPSS (vers. 20, IBM) under Linux. From the 690 events, 22 trials were more than two standard deviations from the mean and were excluded from the ATA analysis as outliers.

### EEG ANALYSIS

EEGLAB (Delorme and Makeig, 2004) under MATLAB for Linux was also used for EEG analysis. For event-related potential analysis, continuous EEG files were re-referenced to an average reference (Lehmann, 1987) and segmented in 3500 ms epochs, including a 3000 ms pre-button-press and 500 ms post-button-press. For a rough pre-selection, all segments with an amplitude of ±100 μV at any channel were rejected. To further minimize the effects due to artifacts, segments with eye artifacts and other abnormal trends were rejected through visual inspection. The trials rejected because of artifacts (32%) were distributed almost equally across the three conditions. RPs were derived by averaging the epochs separately from the three conditions after a DC drift correction based on 3000–2000 ms pre-button-press. For RP onset detection, we used the segmented regression method (Schwarzenau et al., 1998) because it was the most reasonable method when dealing with RP interruptions. In this method, two linear regression lines are calculated. The breaking point of the two regression lines indicates the time of the RP onset. The first regression line of the pre-onset was calculated from the beginning of the segment until the moment at which the regression line began rising and would not become neutral again according to further calculations. From this point on, the second regression line was fitted to the segment that rose to the peak of the RPs. This method proved to be an accurate statistical procedure, as also recommended by Mordkoff and Gianaros (2000). Significant differences in RP onsets were assessed with a jackknife procedure (Miller et al., 1998). RP onset values were entered in an ANOVA, with the *F*-value corrected according to the formula provided by Ulrich and Miller (2001). For visualization purposes, the segments were further reduced in 2500 ms epochs, including 2000 ms pre-button press and 500 ms post-button-press, and the waveforms displayed in the figures were digitally smoothed with a 6 Hz low pass-filter. However, the regression lines were computed using unsmoothed waveforms of the larger segments.

## RESULTS

### REACTION TIME (ANTICIPATION TIMING ACCURACY, ATA)

**FIGURE 2:**
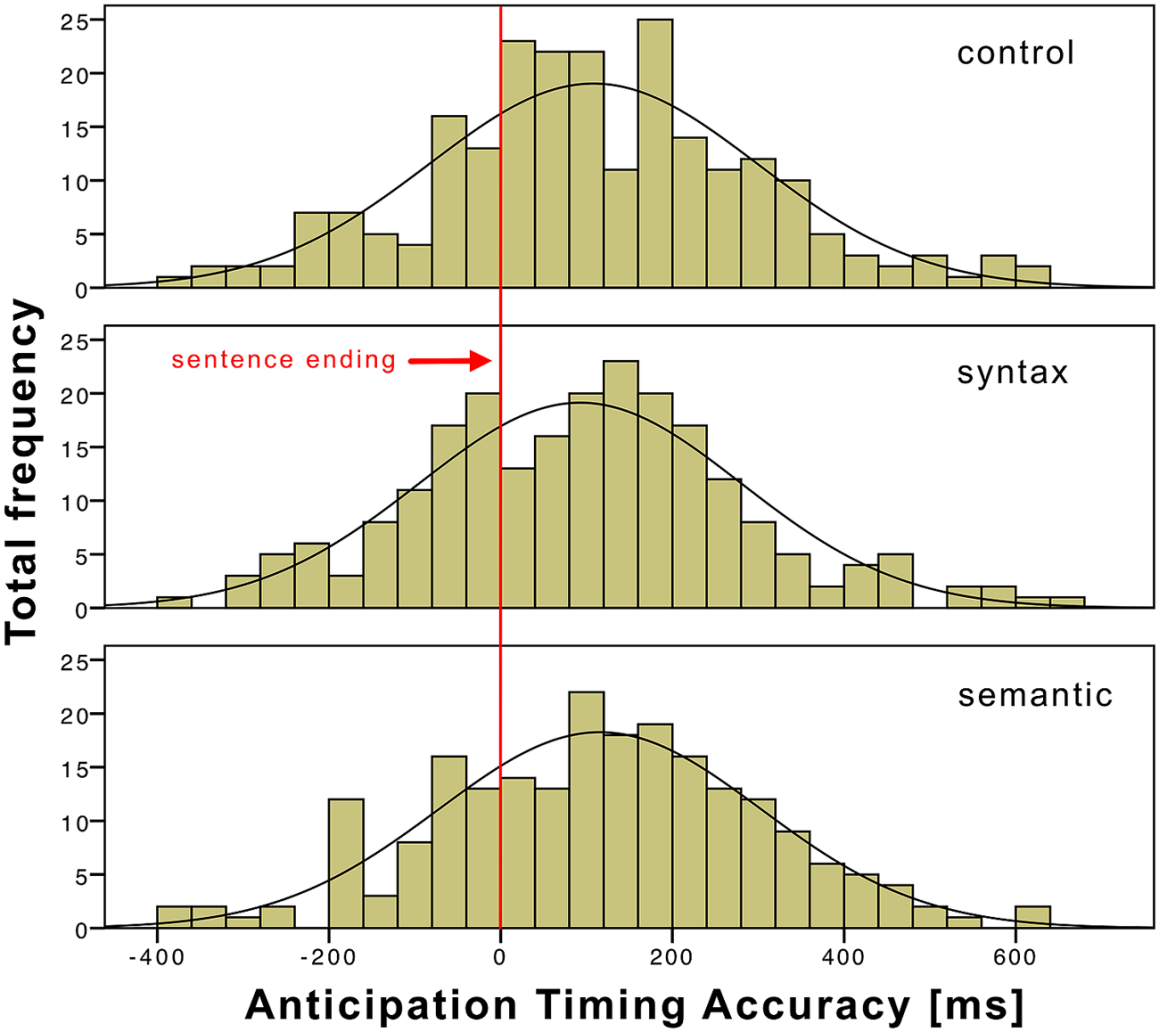
**ATA histogram of the three conditions time-locked (0) to the sentence end and with a bin width of 40 ms**.

Across subjects, 96.8% of the ATAs were within two standard deviations from the mean and were taken into account for statistical calculations and analyzed separately for the three conditions. **Table 3** presents the descriptive statistics of the ATAs for the three conditions. On average, subjects were slowest when responding to sentences with semantic violations (115.6 ms) and fastest when responding to sentences with syntactic violations (92.6 ms). The control condition was in the middle, with an average response time of 108 ms. **Figure 2** shows a histogram of the ATA of the three conditions to illustrate the distributions. A *t*-test did not reveal significant influences of the conditions on the ATA (see **Table 4**).

**Table 3:** ATA descriptive statistics of the three conditions.

Condition	*n*	Mean (ms)	Min (ms)	Max (ms)	SD
Control	228	108.0	-372	639	191.2
Syntactic	225	92.6	-380	654	187.7
Semantic	215	115.6	-390	623	187.8

**Table 4:** *t*-test of the ATA between the three conditions.

	Sum of squares	Df	Mean square	*F*	Sig.
Between groups	60,671.332	2	30335.666	0.850	0.428
Within groups	23,738,844.433	665	35697.510	–	–
Total	23,799,515.765	667	–	–	–

### READINESS POTENTIAL (RP)

**FIGURE 3:**
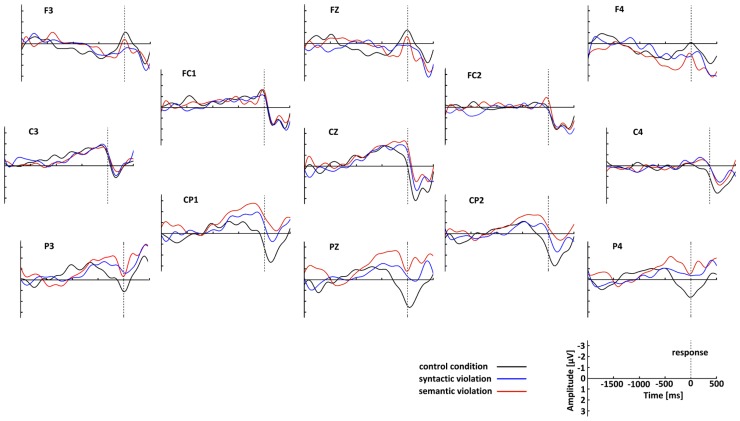
**Grand-average waveforms of the response-locked ERPs separately for the three conditions.** The control condition is plotted in back, syntactic violations in blue, and semantic violations in red. The top of the figure corresponds to the anterior aspect of the head. The negative is plotted up.

**FIGURE 4:**
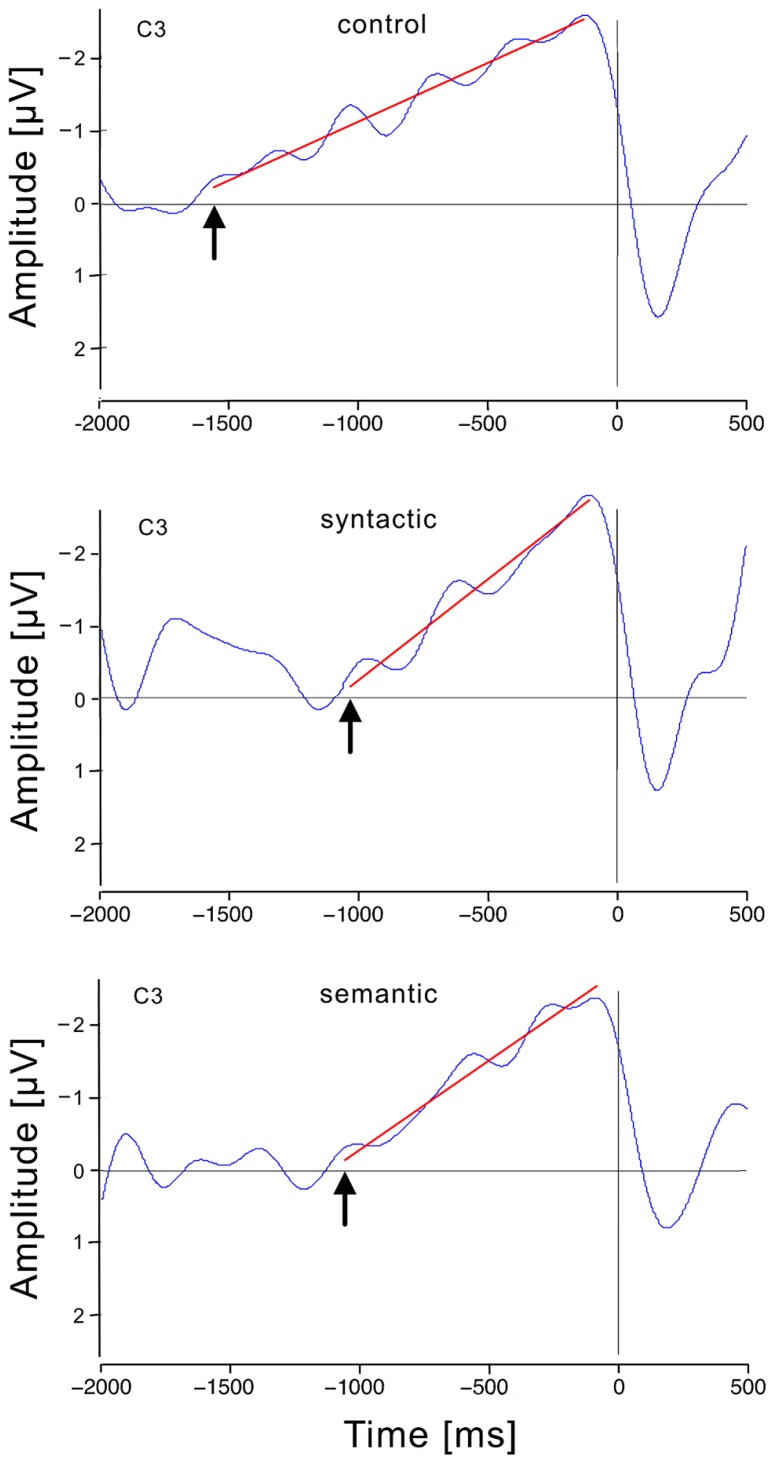
**Response-locked event-related brain potentials at the electrode position (C3) for each condition.** Time 0 is the moment of the responses in this graph, which shows the smoothed RP (blue), and the regression line from the breaking point to the RP peak (red). The breaking points are indicated by arrows.

After artifact rejection, 157 epochs from the control, 158 from the syntax and 154 from the semantic condition from 25 participants formed the grand averages. **Figure 3** shows that the RP in the control condition was, as expected, solid at C3, and further investigation was performed with this electrode. The RP was computed from grand average ERPs at C3 for each condition. RPs displayed a slow negative-going waveform, peaking at approximately 100 ms before response execution. **Figure 4** shows the grand average RP of the three conditions at the electrode position C3.

**FIGURE 5:**
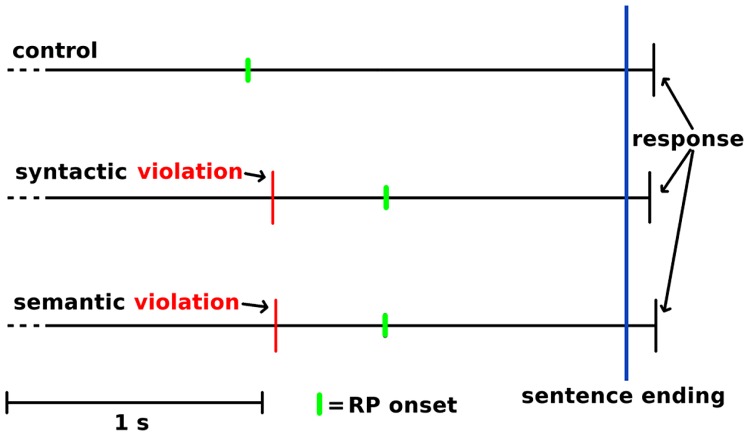
**Illustration of schematic temporal sequences of the sentence-internal violation, RP onset and the response after sentence ending.** Time-locked to the sentence end at the blue vertical line, with the violation-endings indicated by the red narrow lines, and RP onset indicated by the green lines.

The RP onset started at 1560 ms before button-press in the control condition, at 1030 ms in the syntactic condition and at 1060 ms in the semantic condition. There was a significant main effect of condition on the RP onset to button-press interval [*F*_corrected_(2, 72) = 9.766, *p* < 0.01]. Further *post hoc* comparisons revealed a significant RP interruption of 530 ms in the syntactic condition (*M* = 1030, SD = 32.2) compared with the control condition (*M* = 1560, SD = 40.3) and in a significant RP interruption of 500 ms in the semantic condition (*M* = 1060, SD = 34.5) compared with the control condition. The 30 ms difference between the two violation conditions was not significant. To calculate the RP onset to sentence-end interval, the ATA was subtracted from the RP onset to button-press interval. This resulted in an RP onset to sentence-end interval of 1452 ms for the control condition, 937 ms for the syntactic condition and 944 ms for the semantic condition. In addition, the condition had a significant main effect on this interval [*F*_corrected_(2, 72) = 10.694, *p* < 0.01]. *Post hoc* comparisons revealed a significant difference of 515 ms between the control condition (*M* = 1452, SD = 144.7) and syntactic condition (*M* = 937, SD = 136.1) and a significant difference of 508 ms between the control condition and semantic condition (*M* = 944, SD = 129.3). The difference of 7 ms between the syntactic and semantic condition was not significant. **Figure 5** illustrates how the RPs provided us with a better view of the temporal processes for turn-end anticipation and response preparation, compared with behavioral ATA measurements.

## DISCUSSION

Our behavioral ATA results (see **Table 4**) indicate no significant influence of syntactic or semantic sentence violations on turn-end anticipation. However, similar ATAs between conditions fail to reflect the operation of different anticipatory performances, as compensated by short motor-preparation in late anticipation and longer motor-preparation in early anticipation. Although our EEG signatures display clear RP characteristics with a slow negative-going waveform with a peak at approximately 100 ms before response execution, it is possible that they contain some contingent negative variation (CNV) components. Nevertheless, this does not harm the interpretation of our observations. In our response-locked paradigm, both the RP onset and CNV after a warning are considered to be activations caused by preparation. In RPs, the latency of the onset as well as its peak can both vary differentially. Falkenstein et al. (1994), for example, manipulated the complexity of a task by the number of response alternatives. In their results, the degree to which the RP onset was delayed was comparable to the delay of a component linked to the central decision process, whereas the peak of the RP was much more delayed. They concluded that the RP onset was more time-related to central decision processes, whereas the RP peak was more closely related to the actual response. Furthermore, RPs can either be stimulus-locked or response-locked. In both cases, the RP starts at the beginning of motor programming (Masaki et al., 2004). As we attempted to define the moment at which turn-end anticipation is completed and motor programming begins but have no specific moment for the stimulus-locked method in our paradigm, we used the response-locked method. In an EEG experiment, Leuthold et al. (1996) found that a response-locked RP interval is shorter for informative than for non-informative advance information, whereas the offered response speed increased with the amount of advance information. That is, shorter RPs occurred along with shorter RTs, which indicates that the stimulus-RP onset interval remained similar. In our experiment, the ATAs were similar, whereas the RP onsets were different. If we assume that premotor processes and motor preparation are serial processes and ATAs are similar, then turn-end anticipation is finished earlier in early RP onset and later in late RP onset.

An alternative to the assumption of serial processes is the possibility of partial parallel processing. From our RP results in the control condition, we can see that RP onset took place before the violation in the other two conditions. Up to the violation, all conditions consisted of the same text. Therefore, pre-violation RP onset, which was interrupted by the violations, cannot be ruled out. Freeman et al. (2011) reported that an RP interruption is closely linked to continuous neural interactivity between the cognitive and motor systems. Their results revealed that the perceptual-cognitive processing of another’s face is immediately and continuously shared with the motor cortex, whereas the motor cortex takes those results and simultaneously prepares for action. They concluded that the brain begins planning to act on information extracted from another’s face well before it has completely finished interpreting the stimuli. Furthermore, De Jong et al. (1990) found evidence that central response activation processes, as reflected by the RP, can be interrupted and a response can be inhibited at any time during its activation. Regarding these results, incremental turn-end anticipation could have a continuous influence on response preparation in turn-end anticipation.

When using this electrophysiological measure and an RP onset occurring 1452 ms before the sentence end, our results support the general model of turn-end anticipation (Sacks et al., 1974) rather than the reaction model (Heldner and Edlund, 2010). In a meta-analysis on word production, Indefrey and Levelt (2004) stated that experiments have demonstrated that it takes at least 600 ms from the mental concept to articulation. According to our results of RP onset of 1452 ms before an intact sentence ends, not only the 120 to 320 ms pre-activation to word production from Kuriki et al. (1999) but also the 600 ms from the mental concept to articulation fit into this interval. This only holds if the mental concept for the next utterance begins to form at least partially while still listening to intact sentences.

Furthermore, the major finding of this study is the dissociation between the response itself measured by the behavioral reaction time-based ATA and the preconscious RP measure of the response preparation. Our results indicate that the RP can be used to determine whether syntactic or semantic violations influence response preparation in turn-end anticipation. Compared with intact sentences, the syntactic and semantic violations had a significant influence on the RP onset during turn-end anticipation. Thus, both violation types appear to have a significant influence on turn-end anticipation and neither syntax nor semantics can be rejected as a necessary factor for turn-end anticipation. Another possibility is that one type of violation causes problems on the other type of processing level. Pickering and Garrod (2007) have argued that rapid comprehension is enabled by a production-based emulator that enables listeners to imitate what they have heard at various linguistic levels. Such imitation allows the production system to make predictions about upcoming words, grammatical categories and meanings. Furthermore, Pickering and Garrod (2004) assume in a model that as dialog proceeds, interlocutors align their linguistic representations at many levels, including the syntactic and semantic levels. Their model assumes that alignment at one level leads to further alignment at other levels. In the case of an error, there is a direct relationship between other-repair processes in dialog and self-repair processes because the other-monitoring process is directly comparable to the self-monitoring process. Assuming syntactic analysis is unaffected by semantic integration problems, whereas semantic integration is more difficult in the presence of a syntactic processing problem (Hagoort, 2003), it should be considered that both types of violations can result in semantic integration difficulties. Van Berkum et al. (2005) noted that anticipation of upcoming words is a pervasive aspect of ordinary language comprehension, which affects several levels of the comprehension systems, whereby syntactic and semantic processes already interact at an earlier level as morpho-syntactic parsing and semantic integration processes run in parallel (Palolahti et al., 2005). Thus, the RP onset delay in sentences with a syntactic violation could be caused by processing problems on two levels (syntactic processing problems and semantic integration problems), but RP onset interruption in sentences with a semantic violation could be caused by processing problems on only the semantic level, without syntactic processing problems. Independently, it is not even clear that the influence of a syntactic or semantic violation provides information on the necessity of these intact factors for successful language comprehension and turn-end anticipation. Considering the basic assumption that every utterance consists of a correctly formulated meaning, a syntactic or semantic violation might be differently processed compared with the missing syntactic or semantic information. However, the RP results from this study provide evidence that both syntax and semantics are factors that influence turn-end anticipation.

In view of the liability of a button-press in this paradigm, McArdle et al. (2009) highlighted that speech RP is similar to the RP preceding limb movements, as the RP prior to speech is time-locked to the earliest articulatory movement. Thus, even though a button-press is not the natural reaction during turn-end anticipation, it generated plausible differences in RP onsets, as expected.

## CONCLUSION

If participants indicate their anticipated expectation of an end of turn with a button-press, the response-related preparatory motor programming (RP) can be used as a preconscious measure for the time course of this anticipation. In turn anticipation for intact utterances (control), a response-related RP onset of 1452 ms before the turn end provides strong evidence for an early turn-end anticipation rather than reaction to a completed utterance. The minimal syntactic or semantic violations of sentences used in this experiment caused delayed RP onsets but similar behavioral responses. This suggests different response preparations, which are not reflected in the behavioral ATA. The possible different influence of syntactic or semantic information on the anticipation performance suggests that both syntactic and semantic incongruence likewise affect turn-end anticipation processes.

## Conflict of Interest Statement

The authors declare that the research was conducted in the absence of any commercial or financial relationships that could be construed as a potential conflict of interest.
